# Effect of a 16-week multi-level classroom standing desk intervention on cognitive performance and academic achievement in adolescents

**DOI:** 10.1038/s41598-022-18248-y

**Published:** 2022-09-01

**Authors:** Danilo R. Silva, Daniel G. S. Machado, Fernando Pinto, Pedro B. Júdice, Cláudia S. Minderico, Paul J. Collings, Edilson S. Cyrino, Luís B. Sardinha

**Affiliations:** 1 Department of Physical Education, Federal University of Sergipe, Avenida Marechal Rondon, s/no, Rosa Elze, São Cristóvão, SE CEP 49100-000 Brazil; 2 Faculty of Health Science, Universidad Autónoma de Chile, Santiago, Chile; 3 Research Group in Neuroscience of Human Movement (NeuroMove), Department of Physical Education, Federal University of Rio Grande do Norte, Natal, RN Brazil; 4 Department of Social Sciences, Eça de Queiros Secondary School, Lisbon, Portugal; 5 CIDEFES, Faculty of Physical Education and Sport, Lusófona University, Lisbon, Portugal; 6 Exercise and Health Laboratory, CIPER, Faculdade de Motricidade Humana, Universidade de Lisboa, Lisboa, Portugal; 7 Bradford Institute for Health Research, Bradford NHS Foundation Trust, Bradford, UK; 8 Metabolism, Nutrition, and Exercise Laboratory, State University of Londrina, Rod. Celso Garcia Cid, km 380, Londrina/PR, 86057-970 Brazil

**Keywords:** Paediatrics, Public health, Weight management

## Abstract

The replacement of traditional classroom desks for active-permissive desks has been tested to reduce sitting time during classes. However, their impact on other domains is still unclear. We aimed to verify the potential effects of a classroom standing desk intervention on cognitive function and academic achievement in 6th-grade students. This was a controlled trial conducted with two classes [intervention (n = 22) and control (n = 27)] from a public school in Lisbon, Portugal. The intervention was carried out for 16 weeks and consisted of multi-level actions (students, parents, and teachers) centered on the implementation of standing desks in the intervention classroom. The control group had traditional classes with no use of standing desks or any other interference/action from the research team. Pre- and post-assessments of executive functions (attention, inhibitory function, memory, and fluid intelligence) and academic achievement were obtained. No differences between groups were found at baseline. Both groups improved (time effect) academic achievement (*p* < 0.001), memory span (*p* < 0.001), and inhibitory function (*p* = 0.008). *Group versus time* interactions were observed regarding operational memory (intervention: + 18.0% and control: + 41.6%; *p* = 0.039) and non-verbal fluid intelligence (intervention: − 14.0% and control: + 3.9%; *p* = 0.017). We concluded that a 16-week classroom standing desk intervention did not improve cognitive performance or academic achievement more than the traditional sitting classes.

*Trial registration*: ClinicalTrials.gov Identifier (NCT03137836) (date of first registration: 03/05/2017).

## Introduction

The extant literature has shown that the positive effects of regular physical activity on different aspects of health are indisputable. Thus, specific amounts of moderate-to-vigorous physical activity have been recommended by major health organizations. However, an increasing body of evidence has shown that sedentary behavior, which is different from physical inactivity, also has detrimental effects on health parameters^1^. Sedentary behavior is defined as any waking behavior characterized by tasks with an energy expenditure ≤ 1.5 metabolic equivalents, while in a sitting, lying, or reclining posture^2^. Investigations have shown that sedentary behavior impacts postprandial levels of blood glucose, insulin, and ambulatory blood pressure^3–5^. Interestingly, these negative effects may be reversed with interventions involving regular breaks in sedentary behavior as simple as standing up, walking, or cycling at regular intervals (e.g., every 20 or 30 min)^3–5^.

Besides the negative effects of sedentary behavior on physical health, it has also been suggested that sedentary behavior may be harmful to mental and cognitive health^6,7^. In this regard, physical activity and exercise have consistently been shown to provide adaptations in brain function and structure across the lifespan^8–11^. It has been suggested that mechanistic paths of physical activity and sedentary behavior converge in several places supporting the idea that sedentary behavior could negate the positive effects of exercise (Voss et al.^6^). For instance, Wheeler et al. proposed that the negative effect of sedentary behavior on mental health and cognition is via poorer glycemic control (i.e. higher glycemic variability) and lower blood flow provided to the brain.

Studies have shown that sedentary behavior is negatively associated with cognitive performance and/or academic achievement during adolescence (10 to 19 years according to the World Health Organization^12^)^13–15^, indicating that this age is a sensitive period for cognitive adaptations. Given that the patterns of behavior early in life may shape future behavior, health, and cognitive outcomes, understanding how sedentary behavior affects cognitive health and ways to reduce its impact during adolescence is of particular interest. Therefore, it seems reasonable to assume that not only increasing physical activity but also decreasing the time spent in sedentary behavior may benefit mental health and, thus, efficient interventions that target a reduction in sedentary behavior are warranted^16^.

During adolescence, the more common reference to sedentary behavior is thought to be screen time (e.g., TV, video game, computer, smartphones)^17^. However, sedentary behavior constitutes a broader range of activities that can occur during leisure time (e.g., at home, public spaces, travel), while commuting (e.g., private, or public transport), or at school (e.g., recess, classroom time). In general, an adolescent spends around 8 to 10 h per day in sedentary behaviors^18^, and the educational system can be seen as a major promoter of this harmful behavior. Considering that school-aged children sleep around 8 to 10 h per day, half of their waking time is spent at school; and this time is mostly sedentary (total ~ 60%; in classroom ~ 90%)^19^. In other words, of the approximately 8.5 h that children spend engaged in sedentary behavior, 4.8 h (57%) occurs at school. Thus, the school and, especially, the classroom environment constitutes a promising setting for interventions aiming to reduce overall sedentary behavior in youth.

Among the initiatives aimed to reduce sedentary time at school, the replacement of traditional desks for standing desks has been the most effective intervention^20^. This strategy seems to reduce sitting time during classes with no compensatory effects on other domains (e.g., commuting or leisure activities)^21^, but more investigation is needed. Additionally, some commercial classroom standing desks also allow moving around and working in collaborative groups, which can provide more dynamic classes, improving engagement, attention, the flow of ideas, and memory^22,23^. For instance, Mehta et al.^24^ found significant improvement in executive function and working memory in high school students as well as changes in prefrontal cortex activation during the cognitive task after an intervention with a standing desk. In fact, two recent systematic reviews concluded that there is some evidence that active/standing desks could have positive effects on some measures of cognitive performance and/or academic achievement in school-aged children and adolescents^25,26^. However, both systematic reviews emphasized that their conclusions were based on heterogeneous and low-quality studies and that results should be confirmed in randomized controlled trials of better quality^25,26^.

Therefore, good quality trials could shed some light on whether interventions targeting decreases in sedentary behavior in the school classrooms could improve cognitive performance and academic achievements in school-aged children/adolescents. Thus, the current investigation aimed to assess the effects of a 16-week multi-level classroom standing desk intervention on cognitive function and academic achievement in adolescents. Considering the effects of other types of intervention with respect to breaking sitting time in the classroom and effects on cognitive and academic skills^27,28^, it is expected that classroom standing desks will provide a relevant opportunity for improving cognitive health and academic outcomes.

## Methods

### Design and sample

The ERGUER/Portugal project was a cluster-controlled trial conducted in the school setting. For convenience, we selected two sixth-grade classes from a large public school located in central Lisbon. Students from both classes were invited to participate and those aged between 11 and 13 years old who provided parental written informed consent were included in the study (mean ± standard deviation of age: 11.7 ± 0.5 years). Sample characteristics are presented in Table 1. Classes were designated as the intervention group or control group. The intervention consisted of a multi-level standing desk trial performed during classroom time for 16 weeks with two assessment time-points (pre and post intervention). The control group had traditional classes with no use of standing desks or any other interference/action of the research team. This project was approved by the Portuguese Ministry of Education (n.º 0531600001) and the Faculty of Human Kinetics Ethics Committee (n.° 9/2017), and methods were performed following the Declaration of Helsinki.

**Table 1:** Baseline characteristics of the participants.

	Intervention group (n = 22)	Control group (n = 27)	*p value*
Chronological age, years	11.8 ± 0.4	11.6 ± 0.5	0.144
Female	10 (45.5%)	16 (59.3%)	0.336
Caucasian	21 (95.5%)	26 (96.3%)	0.856
Peak height velocity, years	− 1.05 ± 1.15	− 0.93 ± 1.05	0.704
Body mass index, kg/m^2^	19.7 ± 3.0	18.9 ± 3.4	0.154
Academic achievement, total score	26.5 ± 5.0	27.0 ± 4.5	0.717
**Corsi block test**			
Span, level	5.4 ± 1.0	5.3 ± 0.6	0.775
Total score	44.4 ± 15.2	40.6 ± 9.6	0.501
**Stroop test**			
Congruent phase			
Reaction time, ms	1697.5 ± 230.0	1638.4 ± 186.0	0.325
Accuracy, %	97.7 ± 3.8	98.8 ± 3.0	0.287
**Neutral phase**			
Reaction time, ms	1781.1 ± 252.7	1768.9 ± 227.6	0.984
Accuracy, %	98.5 ± 3.3	98.5 ± 4.0	0.802
**Incongruent phase**			
Reaction time, ms	2114.2 ± 341.4	2184.2 ± 227.2	0.278
Accuracy, %	94.7 ± 7.5	91.4 ± 14.3	0.608
**d2 test**			
Accuracy, %	71.1 ± 29.1	63.7 ± 30.8	0.332
**Raven matrices test**			
Standard score, percentile	75.8 ± 25.3	68.7 ± 25.4	0.412

### Intervention procedures

The intervention took place over 16 weeks and comprised of physical and social environmental changes. The *physical environment* was modified by exchanging all traditional seated classroom desks for the LearnFit^®^ Adjustable Standing Desk (Ergotron, USA). In addition to enabling postural changes, these desks are portable and thus expand the possibilities of class-based movements. The *social-environmental* component targeted family support through three specific meetings. In the first meeting, we explained the rationale and components of the study, invited students to participate, and collected written informed consent. The second meeting served to update the parents/guardians about the classroom work and to collect prior perceptions and suggestions to improve pedagogical strategies and maintain motivation related to the intervention. In the final meeting, the main results of the intervention were presented, individualized reports discussed, and further perceptions of parents/guardians about the intervention were collected. An *educational* component entailed six training sessions performed with schoolteachers. The first session involved the dissemination of study information including the project rationale; teachers were then required to present perceived barriers/difficulties and good practices for group discussions to take place (next four sessions). In each session, teacher perceptions about the effectiveness of the intervention were collected. The sixth and final sessions were used to present the results and collect concluding perceptions from teachers. Teachers that attended sessions received professional credits for carrier progression. The information gathered from both parents and teachers was used to continually modify the intervention. Furthermore, peer-to-peer teacher recommendations, such as adopting a U-shaped arrangement of classroom desks, were promoted as examples of best practices that teachers were encouraged to replicate. Further details of the intervention are described elsewhere^21^.

### Measures

#### Descriptive information

Body mass index (BMI, kg/m^2^) was calculated from measurements of weight (nearest 0.1 kg) and height (nearest 0.1 cm). Somatic maturation was estimated by peak height velocity calculated from trunk-encephalic height (measured with a 50 cm bench)^29^. All the measurements were conducted by the same assessor, who had experience with anthropometric procedures. Information on gender and race/skin color (self-reported) was also collected.

#### Academic achievement

Details regarding participant academic achievement were obtained by routine school evaluations (similar to Standard Attainment Tests in England). Home language (Portuguese), mathematics, natural science, foreign language (English), history, visual education, music, and technology grades were collected before (at the end of the first period) and after the intervention period (at the end of the academic year). Student attainment for each subject ranged from 1 (very poor) to 5 (very good). For analyses, all subject marks were summed (total score out of 40).

#### Cognitive performance assessment

Four tests that assess different components of cognitive function were performed:

- *Corsi block-tapping*—a computerized version of the test was used to assess visuospatial working memory^30^. The test involves memorizing the order of nine cubes that flash on a screen. The task starts with two flashes and the difficulty increases progressively. There are two trials for each block-number sequence, and the test is finished when the participant fails to reproduce the correct pattern of cubes flashing twice in the same block number. The test span and the total score were adopted as performance indicators;
- *Stroop Color word test*—a computerized version of the test was used to assess executive function, particularly inhibitory control^31^. This test was composed of three phases (12 trials each) that require participants to choose the correct color name or color ink. In the congruent phase, participants had to indicate the ink color of a rectangle (ink color and color name of the response were the same). In the neutral phase, only color names in white ink were shown, and subjects had to indicate just the color's name. Finally, in the incongruent phase color's names were shown in different ink colors (e.g., "red" in blue ink). Participants had to indicate ink color and ignore the color name. Reaction time and accuracy (% of correct answers) in each phase were captured as performance indicators;
- *d2 test of attention*—the d2 was performed to assess selective attention and concentration capacity^32^. This test consists of one page with 14 rows each containing 47 interspersed *d* and *p* characters. These characters have one to four dashes above and or below, and participants are required to select all *d* characters with two dashes (e.g., d'', 'd,' or ''d). All *p* and *d* with more or less than two dashes are distracters. The test is time-pressured (20 s for each row), and pauses are not allowed. The total number of correct marks (accuracy) was used as the main score.
- *Raven's Progressive Matrices*—was performed to assess non-verbal fluid intelligence^33^. This test consists of 60 multiple choice questions divided into three progressive phases. For each question, participants had to identify the missing element that completes a pattern. A standard score that reflects correct answers was calculated.

Both the Corsi block-tapping and Stroop color tests were performed, in that order, on a computer after standard instructions and a familiarization attempt. The last two tests were performed manually (paper-and-pencil) in groups of 8–10 participants, respectively. Although the procedures were standardized for the assessments, the tests were performed during different periods of the day.

### Statistical analysis

Descriptive data are expressed in frequencies, means, and standard deviations or 95% confidence intervals. Shapiro–Wilk and Levene tests were used to check the normality and homoscedasticity of the data. Baseline comparisons were made by Student t-tests for independent samples, Mann–Whitney, and Chi-squared tests according to variables characteristics. Generalized estimating equation (GEE) models were used for comparing within and between groups for cognitive outcomes before and after the intervention. Linear and Poisson log-linear models of GEE were performed based on each outcome distribution. Statistical significance was set at a 5% level, and data were processed by SPSS software version 26.0.

## Results

Baseline characteristics of the participants [22 in the intervention group (mean age 11.8 years; 45.5% of girls) and 27 controls (mean age 11.6 years; 59.3% of girls)] are presented in Table 1. There was no drop out during the intervention period and no participant switched groups. Overall, students were mostly Caucasian, had not achieved peak height velocity, and had normal BMI. No baseline differences between groups were observed.

Changes in the main outcomes (academic achievement and cognitive tests) after 16 weeks of intervention are displayed in Table 2. Both groups improved (time effect) academic achievement (*p* < 0.001), memory span (*p* < 0.001), and inhibitory function (*p* = 0.008). A *group versus time* interaction was observed for Corsi block-tapping (*p* = 0.039) and Raven matrices (*p* = 0.017). On the other hand, for the non-verbal fluid intelligence, while the control group did not change, the intervention group showed a trend of reducing the test's standard score.

**Table 2:** Mean changes (95% confidence interval) in academic achievement and cognitive tests after a 16-week standing desk intervention.

	Intervention group (n = 22)	Control group (n = 27)
Academic achievement, total score*	1.95 (0.79 to 3.12)	2.04 (1.26 to 2.81)
**Corsi block test**		
Span, level*	0.32 (− 0.14 to 0.78)	0.89 (0.47 to 1.30)
Total score^†^	**4.68 (**− **4.09 to 13.45)**	**15.52 (8.66 to 22.37)**
**Stroop test**		
Congruent phase		
Reaction time, ms*	− 146.2 (− 249.1 to − 43.2)	− 116.2 (− 180.5 to − 51.8)
Accuracy, pp.	0.76 (− 1.19 to 2.70)	0.00 (− 1.83 to 1.83)
Neutral phase		
Reaction time, ms*	− 77.7 (− 195.8 to 40.4)	− 102.2 (− 193.5 to − 10.9)
Accuracy, pp.	− 4.55 (− 14.3 to 5.18)	0.62 (− 0.95 to 2.18)
Incongruent phase		
Reaction time, ms*	− 70.4 (− 198.5 to 57.6)	− 140.4 (− 249.6 to − 31.1)
Accuracy, pp.	− 3.41 (− 8.20 to 1.38)	2.47 (− 3.51 to 8.45)
**d2 test**		
Accuracy, pp	− 7.4 (− 24.4 to 9.5)	2.9 (− 8.7 to 14.5)
**Raven matrices test**		
Standard score, pp^†^	− **10.5 (**− **23.5 to 2.4)**	**2.7 (**− **4.0 to 9.4)**

Significant values are in bold.

*pp* percentage points.

*p < 0.05 for time.

^†^*p* < 0.05 for time versus group interaction.

**Figure 1:**
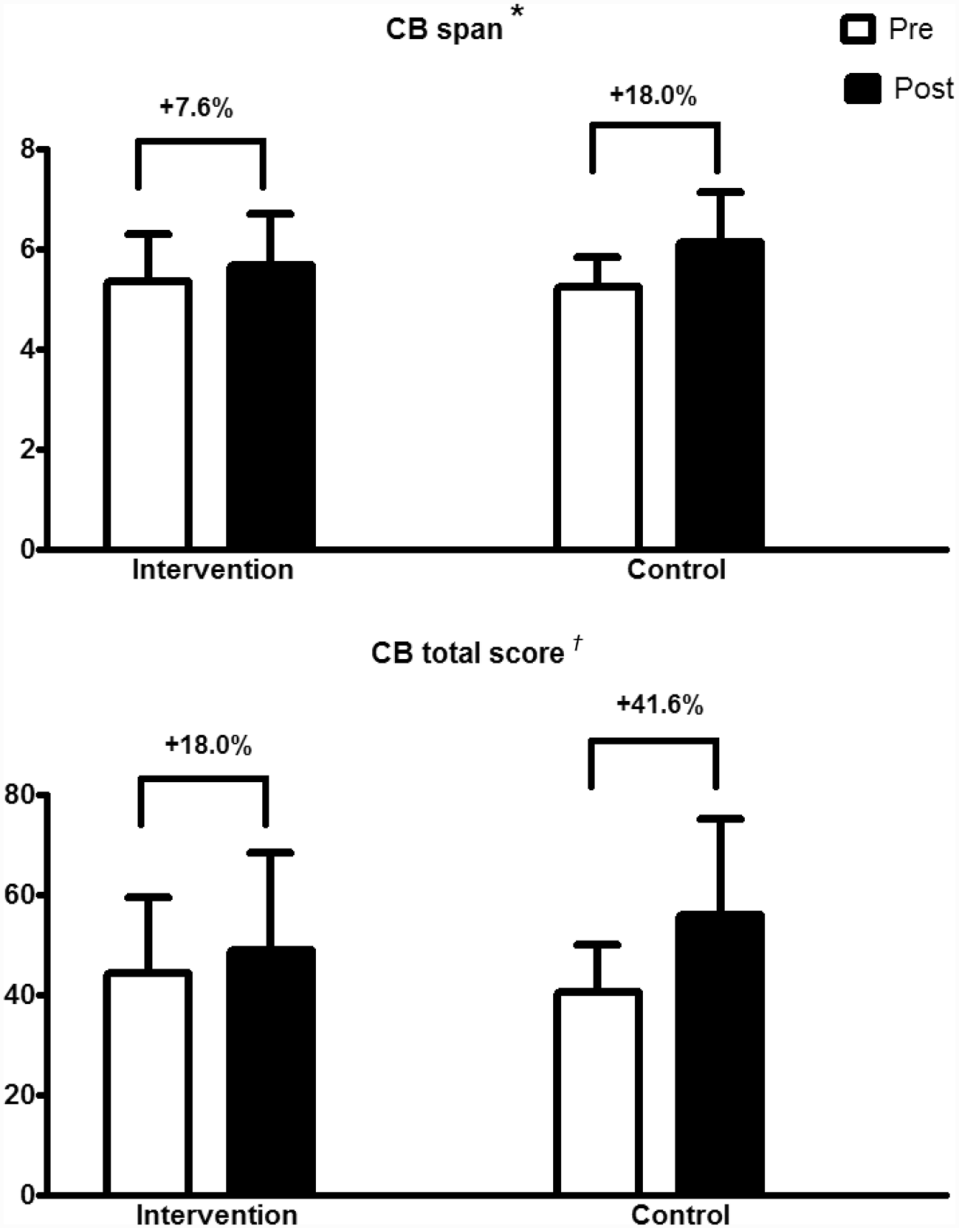
Corsi block-tapping test results (span and total score) pre and post 16 weeks. Data are expressed by the mean and standard deviation. *Note*: *p < 0.001 for time. ^†^*p* = 0.039 for time versus group interaction.

Figure 1 displays the pre and post values of the Corsi block-tapping of the intervention and control groups. While both groups increased the test span (intervention: + 7.6% and control: + 18.0%; *p* < 0.001 for time effect), a group versus time interaction was observed concerning the total score (intervention: + 18.0% and control: + 41.6%; *p* = 0.039).

**Figure 2:**
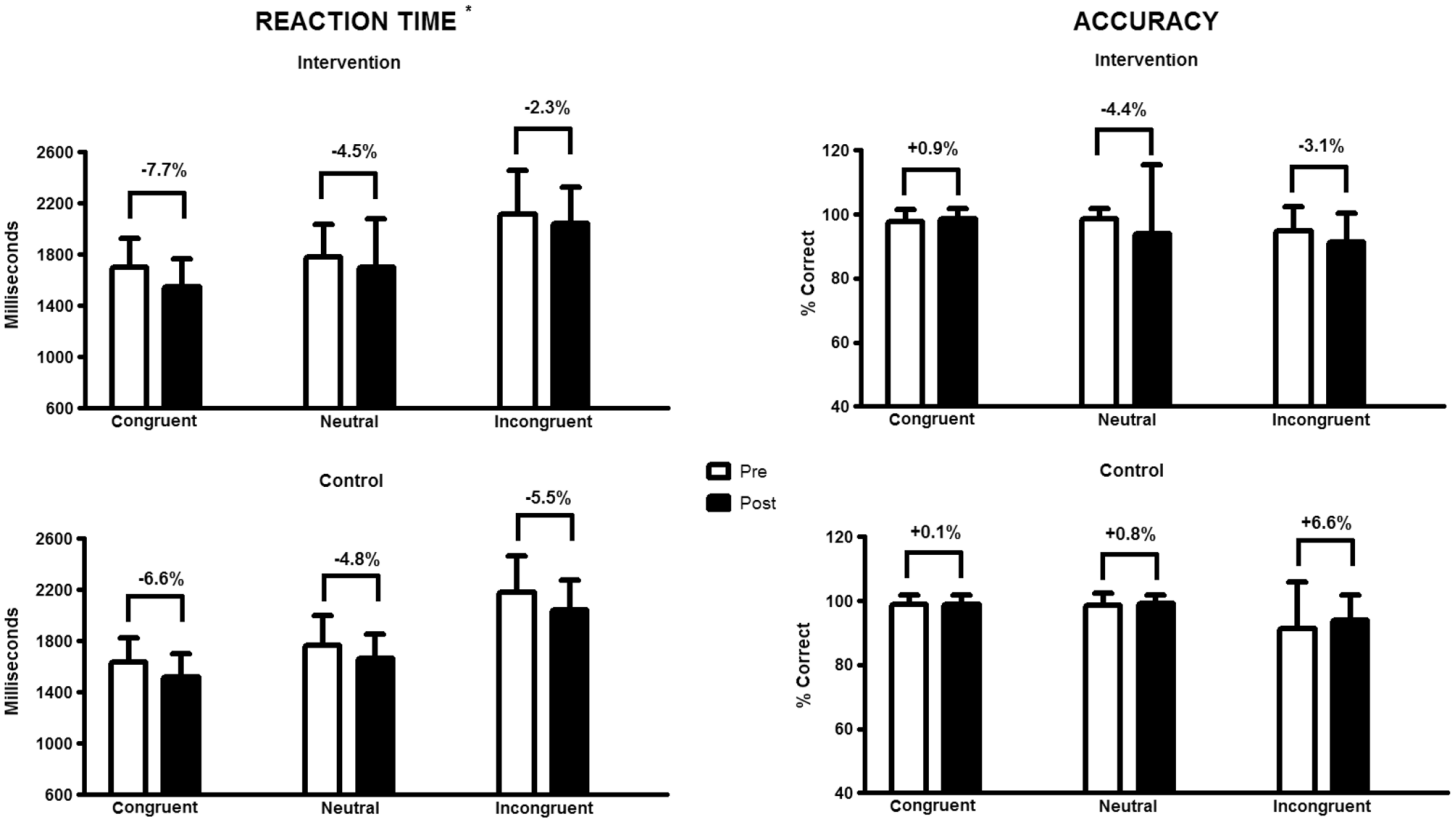
Stroop Color test results (reaction time and accuracy) pre and post 16 weeks. Data are expressed by the mean and standard deviation. *Note*: *p < 0.05 for time in all the phases (congruent: *p* < 0.001; neutral: *p* = 0.011; incongruent: *p* = 0.008).

The results regarding the reaction time and accuracy for the three phases of the Stroop Color test are presented in Fig. 2. We observed a time effect in the reaction time for the congruent (*p* < 0.001), neutral (*p* = 0.011), and incongruent (*p* = 0.008) phases, where both groups reduced, with no difference between them.

**Figure 3:**
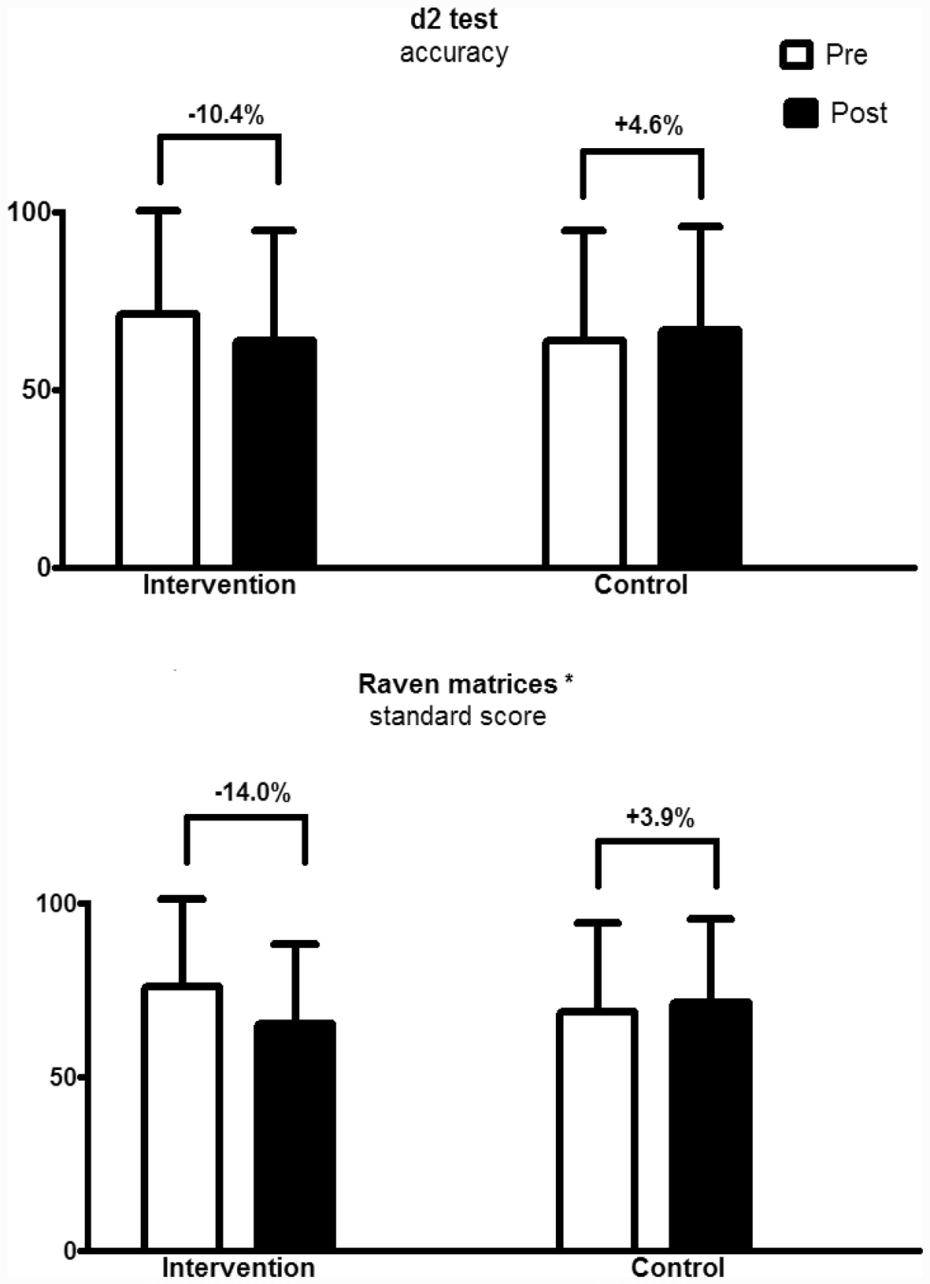
Relative changes in d2 test and Raven matrices performance pre and post 16 weeks. Data are expressed by the mean and standard deviation. *Note*: *Interaction group versus time (*p* = 0.017).

The relative changes in the d2 test and Raven matrices are presented in Fig. 3. While no significant changes were observed for the d2 test, a group versus time interaction was observed regarding the Raven matrices (*p* = 0.017), where the intervention group reduced their score by 14.0% and the control group increased by 3.9%.

## Discussion

The main findings of the current investigation were that a 16-week intervention to reduce classroom sitting time using standing desks did not improve cognitive performance and academic achievement of 6th grade children, in comparison with a control group that continued with the traditional sitting classes. This was one of the first studies to investigate the effects of classroom a standing desk intervention on different cognitive outcomes in adolescents.

Our results oppose previous studies, which have shown positive results of classroom standing desks improving academic engagement, concentration, and cognitive abilities^24,34–36^. However, these studies presented methodological weaknesses (e.g., small sample size, lack of control group), which might explain the conflicting results. For instance, Mehta et al.^24^ were the first to demonstrate the positive effect of stand-biased desks on executive function and working memory capabilities as well as prefrontal cortex activation (during the tests) after 12 months in high-school students. However, their intervention was longer and had no control group, which did not allow for the elimination of possible confounders. On the other hand, our results are in line with a recent systematic review that found the available evidence on the associations between sedentary behavior and executive function to be inconclusive in children and adolescents^13^. This same systematic review found that screen-based sedentary behavior may be negatively associated with cognitive function, which would explain the lack of impact from our intervention. In fact, by introducing the standing desks in the classroom we are only reducing mentally active sedentary behavior and not mentally passive sedentary behavior. Not all types of sedentary behavior seem to be negatively associated with cognitive performance. For instance, while the time spent in sedentary behavior not related to academic skills (e.g., TV/screen viewing) was negatively associated with cognitive performance, sedentary behavior related to academic skills (e.g., reading, doing homework, writing) can be positively associated with cognitive performance and academic achievement in children/adolescents (aged 5–17 years)^13,37,38^.

Two results reached statistical significance, the increase in visuospatial working memory (i.e., Corsi Block-taping), which increased largely in the control group, and fluid intelligence (i.e., Raven matrices), which worsened in the intervention group. The reason for these results are unknown and contrary to our expectation. However, in most school-based interventions it is difficult to control all potential confounders that might influence cognition during adolescence, which is an important transition phase^39^, as well as to guarantee that changes in cognitive function are caused by the postural transitions. As shown in a previous study^21^, our intervention was effective in reducing sitting time and increasing standing time at school with no effects on physical activity outside the school on both weekdays and weekends^21^. Also, no changes were observed in sleep duration (data not shown), which could influence cognition abilities^40^. However, we did not measure other behaviors such as the dietary intake during the intervention, which may be associated with cognitive functioning or even mediate the associations between sedentary behavior/physical activity and cognitive outcomes^41^. This is inherent to research performed in a real environment. Nonetheless, process evaluation is needed to better understand mediators, moderators, and potential links in the causal chain.

Although still controversial, recent empirical data suggests that breaks in sitting time through bouts of at least 10 min of physical activity may improve some executive function and academic skills among youth^27,42,43^. However, it is not clear how these breaks affect specific executive functions^44^. Interestingly, greater benefits were observed among students with lower intelligence quotients and lower initial grades^43,45^, which is especially important given that adolescents who had lower academic performance tend to be more sedentary^46^. Also, the effects of breaks in sitting time can vary according to gender or physical fitness^43^. Here, unfortunately, we have no statistical power to perform sub-group analyses. It is important to mention some previous studies that tested more intensive breaks (through moderate physical activity), while our intervention only led to the replacement of sitting with standing time^21^. To date, even with evidence of the harmful effects of uninterrupted sedentary behavior, there is no consensus upon what should be done during these breaks or the ideal frequency of these breaks^47–52^.

Although the evidence relating to physical activity, exercise, and physical fitness benefits for cognitive function seem clear, the effect of sedentary behavior is not. Different types, durations, and patterns of sedentary behavior have shown mixed results, especially because some sedentary activities can stimulate cognition (e.g., studying, working, playing board games)^53,54^, while others do not (e.g., TV-watching). Our intervention only reduced sedentary behavior at school^21^, meaning that the same academic activities were performed, but standing instead of being seated. We did not observe changes in sitting time outside of school. In addition, no effect of the intervention on daily physical activity measured by accelerometry was found (i.e., step counts, light physical activity, or moderate-to-vigorous physical activity) ^21^, which may explain our results. One can hypothesize that the behavioral modification attained in our intervention (i.e., replacing sitting with standing during classes) may not be enough to improve cognitive outcomes, without changes in the outside of school time or a higher intensity of activity during this sitting time replacement^21^. Thus, future interventions must not only target the school time but simultaneously the extra-school setting.

Considering the potential of the standing desks the ability to move and work in collaborative groups, more dynamic classes are possible. More research about pedagogical work with this new tool is needed to provide varied and enjoyable experiences for students. Potentially, it is not only the reduction of sitting time, but also its interaction with higher levels of physical activity that can optimize cognitive abilities.

An important topic regarding the effects of behavioral interventions on cognition is their applicability. More evident exposures (i.e., physical activity, physical fitness) seem to predict cognitive abilities, but it is still not clear how these abilities are translated to the real world (e.g., general learning, better academic, and productive life)^55^. In our investigation, for example, the potential negative effects observed in some cognitive abilities (pre and post comparisons) were not transferred to academic achievement. Thus, future studies should consider outcomes that are more practical and that better translate to academic achievement. Also, studies involving sedentary behavior must use different types of breaks such as standing, light, moderate and vigorous exercising (e.g., treadmill, bike, elliptical) with different durations for the breaks and time intervals. In the present study, we used the least physiologically demanding break, which was continuously standing.

From a practical point of view, although the results of the present study did not show a clear positive effect of replacing sitting time with standing on cognitive function and academic achievement in adolescents, interventions should be encouraged due to its short- and long-term positive effect on ambulatory blood pressure^4^, glycemic control^5^, and circulating blood insulin^3^. Moreover, lack of physical activity and more sedentary behavior is independently associated with neurodegenerative diseases such as dementia^56^ and mild cognitive impairment^57^. Therefore, it seems reasonable that public health policies must not only promote physical activity but also the reduction of sedentary behavior.

Limitations of the present study include the assessment of only a few domains of cognitive abilities. Although no clear evidence is available on the specific cognitive domains affected by sedentary behavior, we highlight that the tests used in the current investigation are among the most commonly used tests for cognitive assessment in the physical activity research field^58^. The lack of monitoring for possible confounding variables such as sleep or dietary intake as well as the completion of cognitive tests at different periods of the day, should also be mentioned as a limitation. The generalization of our findings must take this into account. The strength of the present investigation includes the presence of a control group, a longer intervention of four months, which comprised physical and social environmental changes, and school teacher training to ensure that both children and teachers' adapted to the new classroom reality. We showed the effects of a classroom standing desk intervention on different domains of executive function and academic achievement, advancing the current knowledge in this field, although more studies are needed (e.g., longer intervention periods and different cognitive outcomes such as focus and knowledge retention). Hillman et al.^55^ highlighted some gaps in the relationship between physical activity, brain, and cognition in childhood. Because this is a relatively recent research field, there are still many gaps to be filled, and further studies should explore the effects of sedentary behavior on the brain and cognition, especially regarding their different contexts, patterns, dose–response, individuality, and sensitivity periods.

We conclude that a 16-week classroom standing desk intervention did not improve cognitive performance or academic achievement in 6th-grade students. The intervention group displayed a lower score than controls on the Raven matrices test. These effects should be interpreted as a whole intervention process more than only postural change and fewer sitting classes. Larger studies, with longer follow-up and incorporating a process evaluation are warranted to clarify the potential mechanisms and long-term effects of standing desks on cognition and academic performance, including their interactions with biological development processes.

## Data Availability

The datasets generated during and/or analyzed during the current study are available from the corresponding author on reasonable request.
